# Multistate Models for the Recovery Process in the Covid-19 Context: An Empirical Study of Chinese Enterprises

**DOI:** 10.1007/s13753-022-00414-5

**Published:** 2022-05-16

**Authors:** Lijiao Yang, Yu Chen, Xinyu Jiang, Hirokazu Tatano

**Affiliations:** 1 School of Management, Harbin Institute of Technology, Harbin, 150001 China; 2 School of Management, Wuhan University of Technology, Wuhan, 430070 China; 3 Disaster Prevention Research Institute, Kyoto University, Kyoto, 611-0011 Japan

**Keywords:** Accelerated failure time model, China, Covid-19, Enterprise recovery process, Multistate model, Recovery state

## Abstract

The Covid-19 pandemic has severely affected enterprises worldwide. It is thus of practical significance to study the process of enterprise recovery from Covid-19. However, the research on the effects of relevant determinants of business recovery is limited. This article presents a multistate modeling framework that considers the determinants, recovery time, and transition likelihood of Chinese enterprises by the state of those enterprises as a result of the pandemic (recovery state), with the help of an accelerated failure time model. Empirical data from 750 enterprises were used to evaluate the recovery process. The results indicate that the main problems facing non-manufacturing industries are supply shortages and order cancellations. With the increase of supplies and orders, the probability of transition between different recovery states gradually increases, and the recovery time of enterprises becomes shorter. For manufacturing industries, the factors that hinder recovery are more complex. The main problems are employee panic and order cancellations in the initial stage, employee shortages in the middle stage, and raw material shortages in the full recovery stage. This study can provide a reference for enterprise recovery in the current pandemic context and help policymakers and business managers take necessary measures to accelerate recovery.

## Introduction

The sudden outbreak and sustained impact of the Covid-19 pandemic has plunged the global economy into recession (McKibbin and Fernando 2021). In this context, the research topic of business resilience has aroused and promoted extensive attention of the academic community. Business resilience basically includes two aspects—resistance and recovery. Resistance refers to the ability of a business to remain at a given functional level at a certain point during the impact of a disaster, emphasizing the measurement of static resilience. Recovery refers to the ability of an enterprise’s business activity to recover from the impact of a disaster, emphasizing the measure of dynamic resilience (Rose 2004; Rose and Krausmann 2013). In the context of Covid-19, static resilience was investigated in terms of factors that accelerate or decelerate enterprise recovery time (Yang et al. 2021). However, the factors are unstable with change in the internal and external environment, although after the initial impact period pandemic control had been normalized in China, and most enterprises then resumed activities (Han et al. 2020). Therefore, studying the determinants and dynamic process of rapid recovery cases among Chinese enterprises can not only deepen the understanding of business resilience in disasters but also provide a reference for promoting the recovery of global enterprises and reducing the cascading effects due to the supply chain interruption among enterprises.

Many studies have identified the determinants of business recovery following disasters (Kachali et al. 2015; Leelawat et al. 2015; Presutti et al. 2016), which are mainly divided into internal and external determinants. Internal determinants are reflected in the vulnerabilities of enterprises, and include enterprise type, size of the workforce, supply and capital constraints, and entrepreneurship (Asgary et al. 2012; Dahles and Susilowati 2015; Battisti and Deakins 2017). External determinants are related to the hazard intensity, such as the intensity of earthquakes and typhoons and the submerging depth of floods (Webb et al. 2002; LeSage et al. 2011), or the severely disrupted social and economic environment following a disaster (Stevenson et al. 2014; Cowling et al. 2020; Adam et al. 2021). As the above-cited literature reflects, the research on post-disaster recovery of enterprises has mainly focused on the overall or static perspective. However, enterprise business recovery is a multidimensional, nonlinear process, which may be affected by different determinants at different stages of recovery (Liu et al. 2021). Therefore, the understanding of the dynamic enterprise recovery process is also essential.

The recovery process of enterprises is usually treated as a whole in previous studies due to the difficulty of measurement. Pathak and Ahmad (2016), for example, revealed that the recovery period of small and medium enterprises (SMEs) that were affected by the 2011 floods in Thailand was one month to 12 months. Yang et al. (2020) reported that the total stagnation and recovery time for the industrial sector in Yuyao, China, was conditioned by inundation water depth under the impact of 2013 Typhoon Fitow. Recently, scholars have focused on the characteristics of a certain recovery stage. Chatterjee et al. (2018) found that the initial destruction of utilities and road infrastructure during the 2015 Nepal earthquake made early recovery of SMEs more challenging. Kajitani and Tatano (2009) assumed a discrete process of recovery of lifeline infrastructure in an earthquake disaster and used a computable general equilibrium model to analyze the economic impact of the disaster on the society in different recovery states. Developing this idea, Liu et al. (2021) modeled the recovery process of the Japanese industrial sector after the 2016 Kumamoto earthquake using a multistate semi-Markov framework.

Previous studies on business recovery after natural hazards and disasters provide clear insights and guidance for this study, but two realistic problems related to the Covid-19 situation remain to be addressed. First, worldwide, Covid-19 is not over yet, and enterprise recovery is unsettled. At present, research on enterprise recovery has difficulty measuring the whole recovery process. Second, the study of economic effects of natural hazards and disasters generally assumes that the internal structure of enterprises does not change significantly, and that the determinants that affect the recovery of enterprises result mainly from disaster-inducing factors—that is, it focuses on the short-term effects. However, Covid-19 will have a long-term impact on the economy, and enterprise recovery is a dynamic process that involves changes in one or more determinants over time. There is a necessity to dynamically analyze the determinants and the process of enterprise business recovery.

The methods used to study the post-disaster recovery process in the literature can be divided into three categories—data-driven models (Chang 2010; Monteil et al. 2020); simulation-based models (Eid and El-adaway 2017; Ghaffarian et al. 2021); and stochastic process models (Lin and Wang 2017; Dhulipala and Flint 2020). However, the dynamic recovery process of an enterprise is composed of a single or multiple events with time-varying covariates (Marshall and Schrank 2014), and none of the current methods can address the time-varying heterogeneity caused by discrete-outcome data gathered over time. In order to fill the gaps in the literature, this study applied a multistate model combined with survival analysis, including a parametric survival analysis model used to analyze the determinants and time length of a discrete recovery period, which solves the difficulty of censored data (from businesses that are not completely recovered), and a multistate semi-Markov process was used to model business recovery. Compared with other methods for describing the post-disaster recovery process, the multistate model with the help of the survival analysis model can estimate the time-varying effect of covariates and can also express the evolution between stages in terms of transition probability. This study contributes to the research field in three ways: (1) This study used the parametric accelerated failure time model to investigate the multiplicative effects of recovery time dependence on time-varying covariates by taking different recovery states of enterprises as the events of interest; (2) The transition probability between different recovery states and the influence of covariates on the transition probability were also evaluated by the multistate model; and (3) The study collected evidence from China, where pandemic control has been normalized and most enterprises have resumed activities, to provide a reference for work promoting the recovery of global enterprises.

## Data and Methods

The empirical data and the survey approach employed in this study are presented in Sect. 2.1, and the relevant definitions and methods are presented in Sects. 2.2 and 2.3.

### Data Description

To answer the research questions, we collected data on the impact of the Covid-19 on enterprises through an online questionnaire survey. A stratified random sampling method was adopted to ensure the representative distribution of sample enterprises. We used the provinces as stratum and randomly selected a certain number of samples from each province according to provincial-level GDP, so as to describe the spatial distribution characteristics of regional development in the provinces. The survey period was from 1 July to 30 September 2020. The questionnaire was distributed and collected with the help of Zhongyan Network Technology Co., Ltd., of Shanghai,^1^ a for-profit professional survey company. In order to ensure the reliability of the data, the survey company communicated with and interviewed the enterprise managers on the enterprises’ post-disaster recovery at any time during the survey. The enterprise name was used as the keyword to match the business information query platform,^2^ to obtain the enterprise attribute information. Since some enterprises did not recover to a certain state during the survey period, namely, no transition of recovery state was observed, we call the data of these enterprises as censored data. The study is based on 750 valid samples, with 367 in non-manufacturing industries, showing 931 observed state transitions, and 383 samples in manufacturing industries, with 969 observed state transitions. Manufacturing was further subdivided into raw materials, processing and assembly, and livelihood-related, while non-manufacturing was subdivided into services and wholesale and retail (Yang et al. 2016).

Descriptive analysis results for all the data are presented in Table 1. The results of the descriptive analysis show the distribution of days after the resumption of business operations in the initial, mid-term, and full stages, and 95% confidence intervals for upper and lower mean in days of operation recovery. The statistical test was two-sided, and a statistically significant *p* < 0.05 was calculated for the model and parameters.

**Table 1:** Descriptive analysis of the data on days of operation recovery of the 750 sample businesses in China

	Non-manufacturing (*n* = 367)			Manufacturing (*n* = 383)		
	Initial recovery	Mid-term recovery	Full recovery	Initial recovery	Mid-term recovery	Full recovery
Mean	35.63	66.24	116.98	33.79	62.85	106.83
Standard deviation	21.54	39.31	68.17	17.86	30.89	54.22
Upper 95% mean	37.84	70.27	123.98	35.58	65.95	112.28
Lower 95% mean	33.42	62.21	109.98	31.99	59.74	101.39
Maximum	100	200	400	100	200	400
Minimum	10	14	21	7	15	18
Censored	0	26	144	0	37	143

### Definitions

This section presents the relevant definitions applied by the model.

#### Recovery State

**Fig. 1:**
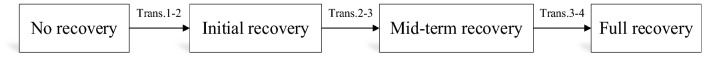
Multistate framework for enterprise recovery from Covid-19 disruption in China. *Trans.* Transition

The recovery state of an enterprise was defined as the proportion of its post-disaster operation (production, sales, and so on) capacity recovered to the pre-disaster normal level of operation. Consistent with previous studies (Kajitani and Tatano 2014; Yang et al. 2016), the recovery state was divided into four categories—no recovery, initial recovery, mid-term recovery, and full recovery (Fig. 1).

- No recovery: The enterprise has not recovered, and is in a stagnant state.
- Initial recovery: The rate of recovery to a normal level of operation is greater than 0 and lower than or equal to 1/3.
- Mid-term recovery: The rate of recovery to a normal level of operation is greater than 1/3 and lower than 2/3.
- Full recovery: The rate of recovery to a normal level of operation is greater than 2/3 and lower than or equal to 1, which represents sufficient recovery.

We consider the recovery of an enterprise as a multistate stochastic process and explored how recovery time varies based on the specific determinants of each state.

#### Transitions

The changes between recovery states are called transitions, which in turn contain information about the dynamics of enterprise recovery. The potential transitions for this study were defined as:

- Trans. 1–2: The enterprise goes from no recovery to initial recovery.
- Trans. 2–3: The enterprise goes from initial to mid-term recovery.
- Trans. 3–4: The enterprise goes from mid-term to full recovery.

Enterprise recovery is a continuous irreversible process, and enterprises that reach the mid-term recovery stage must have experienced the initial recovery process. Transitions between states do not necessarily occur, as they are related to factors such as the damage suffered by an enterprise in the context of the pandemic and the resources available to that enterprise (De Mel et al. 2012). Estimating the transition probability between different recovery states after introducing covariates can provide us with a clearer understanding of the recovery process of an enterprise, which is another focus area for this study.

#### Recovery Time

In previous studies that considered the stagnation and recovery times of enterprises under the scenario of heavy rains and flooding, Yang et al. (2016, 2020) defined recovery time as the time when the entire recovery state ends, focusing on the results of enterprise recovery and ignoring the dynamic changes of the recovery state. In our study, the time required for an enterprise to shift between states was estimated. Therefore, we defined survival time as the time at the end of different recovery states, as the time to state $j$ given the initial state is state $i$. Simultaneously, a set of explanatory variables to explore the covariate effects of recovery time were introduced. These variables are summarized in Table 2.

**Table 2:** Explanatory variables

Variables	Description
Business ownership	The enterprise is state-owned (= 1) or non-state-owned (= 0)
Customer distribution	If the enterprise customers and the enterprise are in the same province (= 1); otherwise (= 0)
Number of employees	The number of employees on the enterprise payroll
Raw material (supply) shortages	For manufacturing or non-manufacturing, the percentage of raw material or supply shortages during the recovery process
Cash flow shortages	The enterprise faces a cash flow shortage (= 1); otherwise (= 0)
Employee panic	If an enterprise faces employee panic, which leads to inefficiency at work (= 1); otherwise (= 0)
Employee shortages	The percentage of employee shortages during the recovery process
Order cancellations	The percentage of orders that have been cancelled compared to previous years

### Methods

The following subsections present the research methods applied in this study, including the accelerated failure time model and the multistate model.

#### Accelerated Failure Time Model

Survival analysis was used to study the expected duration of one or more events before they occur. This type of analysis has been mainly used in pathology studies (Clark et al. 2003; George et al. 2014), but is now gradually expanding to economics, sociology, and other fields (Mikucka 2012; Gémar et al. 2016). Combining survival analysis with multistate models is a new research line (Gran et al. 2015; Carobbio et al. 2020; Zhang and Fricker 2020), especially for enterprise recovery.

In survival analysis, proportional hazard and accelerated failure time models are often used in multivariate analysis (Bradburn et al. 2003a). The difference between them lies in the basic assumptions of the models. The basic assumption of the proportional hazard model is that the effect of the covariates is multiplied with respect to the survival hazard. In contrast, the assumption of the accelerated failure time model is that the effect of the covariates is multiplied with respect to the survival time. Since the assumption of the hazard multiplicative effect is more difficult to satisfy, this makes the accelerated failure time model an effective alternative when this assumption is violated (Bradburn et al. 2003b). Based on the above considerations, the accelerated failure time model was used to evaluate the recovery time of the enterprise.

The accelerated failure time model assumes that the time of an event’s occurrence follows a particular probability distribution, such as exponential, Weibull, log-logistic, or log-normal. The log-normal accelerated failure time model is shown as an example. The probability density, survival, and hazard functions of the log-normal distribution are, respectively, as follows:

1 $$f(t) = (1/t\sigma \sqrt {2\pi } )\exp ( - {(\ln t - u)^2}/2{\sigma^2}),$$

2 $$S(t) = 1 - \Phi \left( {(\ln t - u)/\sigma } \right),$$

3 $$h(t) = f(t)/S(t),$$

where $u$ is the scale parameter, which represents the median of the distribution; $\sigma$ is the shape parameter, which determines the shape of the hazard function; and $\Phi (x)$ is the cumulative distribution function of the standardized normal distribution. The log-normal model defines the relationship between the covariates and parameters by setting $u = {x^T}\beta$ and including the standard deviation, and $\sigma$ as an auxiliary parameter estimated based on the data. The influence of the covariate effects on survival time can be scaled to any fixed value of $S(t)$. With the introduction of covariates, the survival function Eq. 2 can be rewritten in the following form:

4 $$S(t,x) = 1 - \Phi \left( {(\ln t - {x^T}\beta )/\sigma } \right).$$

The above equation can be used to determine whether the recovery time is accelerated or decelerated by the sign of the covariate term. To compare it with the baseline recovery time, we call $\exp ({x^T}\beta )$ the acceleration factor. When $\exp ({x^T}\beta ) > 1$, the recovery time of the enterprise to a certain state is amplified, that is, enterprise recovery is decelerated; alternatively, when $\exp ({x^T}\beta ) < 1$, enterprise recovery time is reduced, that is, enterprise recovery is accelerated.

#### Multistate Model

The accelerated failure time model was used to evaluate the influence of the exploratory variables on the enterprise recovery time of different states. The variables screened by the accelerated failure time model are the key determinants of the change in an enterprise’s recovery state and are introduced into the multistate model to measure the influence of dynamic changes between the recovery states.

The multistate model describes the transition between different states over time, which makes it a suitable choice for modeling the enterprise recovery process. A detailed description of the multistate model can be found in several related studies (Andersen and Keiding 2002; Putter et al. 2007; Titman and Sharples 2010). Critical concepts applied in this multistate model are as follows:

Let $X{(t)_{t \geqslant 0}}$ represent a stochastic process in a finite state space, $S = \left\{ {1,2,3, \ldots ,N} \right\}$, where $X(t)$ is the state of the enterprise at time $t$. An event is defined as a transition between model states, as shown in Fig. 1. An important measure of the dynamic changes between states in a multistate model is the transition probability, defined as:

5 $${P_{ij}}(s,t) = P\left( {{X_t} = j|{X_s} = i,{X_{S^- }}} \right),s \leqslant t,$$

where ${P_{ij}}(s,t)$ represents the probability of an enterprise moving from state $i$ to state $j$ at time interval $\left[ {s,t} \right)$; and ${X_{s^- }}$ includes the cumulative historical information before time $s$. The instantaneous hazard rate of an enterprise transitioning from state $i$ to state $j$ at time $t$, also called transition intensity, is defined as:

6 $${\lambda_{i,j}}(t) = \mathop {\lim }\limits_{\Delta t \to 0} \left( {P\left( {{X_{(t + \Delta t)}} = j|{X_t} = i} \right)/\Delta t} \right),i \ne j$$

The transition probabilities can be expressed in matrix form as:

7 $$\hat P(s,t) = {\prod_{u \in (s,t]}}(I + d\hat A(u)),$$

where $I$ is the unit matrix and $\hat A(u)$ the cumulative transition intensity matrix estimated using the Nelson-Aalen estimator (Aalen et al. 2008). To capture the variation of transition probabilities under different influences, we obtain the transition probability matrix, conditional on the restricted covariate vector $Z$, as:

8 $${\hat P_Z}(s,t) = {\prod_{u \in (s,t]}}\left( {I + d{{\hat A}_Z}(u)} \right),$$

where ${\hat P_Z}(s,t)$ and ${\hat A_Z}(u)$ are the estimated transition probability and cumulative transition intensity matrices for the specific covariates, respectively.

Multistate models usually require that stochastic process $X(t)$ follows the Markovian assumption that the occurrence of the next state and the time of occurrence only depend on the current state. However, the Markovian assumption condition ignores the historical information of previous states, thus being harsh and limiting. In this study, we loosen the Markovian assumption restriction and assume that $X(t)$ is semi-Markovian. The recovery of enterprises under major disaster scenarios is a long process, which encompasses several different states as market and internal conditions change. This assumption is reasonable here, since a previous state in enterprise recovery may significantly affect the progress to a subsequent state (Blackman et al. 2017). For instance, enterprises that initially recover better may also recover faster in the middle and later stages.

## Empirical Results

This section presents the selection result of accelerated failure time models based on empirical data, then describes the covariates that significantly affect the different stages of enterprise recovery, and presents the results of the analysis of the effects of covariates on the recovery state transition probability of enterprises.

### Selection of Accelerated Failure Time Model

The flexibility of parametric accelerated failure time models makes the selection of an appropriate distribution crucial. As such, Akaike information criterion (AIC) values are used to select accelerated failure time models following different distributions (Bradburn et al. 2003b); AIC values weigh the goodness of fit of a model against its complexity and measure the degree to which the model explains the data. A low AIC value indicates a better fit of the model to the data. The candidate distributions applied to the accelerated failure time model are the exponential, Weibull, log-normal, and log-logistic distributions. The AIC values of the models with four distributions for the recovery time of enterprises are listed in Table 3. Based on the AIC values method, we chose the log-normal distribution that provided the best fit with the enterprise recovery data. To maintain theoretical consistency, a log-normal accelerated failure time model was used to analyze enterprise recovery time and its determinants.

**Table 3:** Akaike information criterion (AIC) values for the different distributions for industries

Industry	Trans.	Distributions			
		Exponential	Weibull	Log-normal	Log-logistic
Non-manufacturing	1–2	3359.83	3203.32	**3179.65**	3204.33
	2–3	2997.86	2928.41	**2876.40**	2879.14
	3–4	2331.02	2324.55	**2269.39**	2271.56
Services	1–2	2056.12	1975.18	**1950.08**	1964.13
	2–3	1803.10	1766.38	1724.26	**1723.95**
	3–4	1410.21	1408.57	1370.66	**1369.40**
Wholesale and retail	1–2	981.55	**924.68**	935.50	942.70
	2–3	892.43	875.02	**871.33**	874.80
	3–4	601.86	601.73	**592.14**	595.59
Manufacturing	1–2	3455.60	3226.17	**3214.08**	3235.25
	2–3	3087.66	2962.90	2953.16	**2952.75**
	3–4	2418.13	2385.08	2344.39	**2340.71**
Livelihood-related	1–2	548.76	515.19	**511.49**	515.64
	2–3	491.96	**471.52**	473.77	474.90
	3–4	386.63	379.24	**369.93**	370.19
Processing and assembly	1–2	1170.22	1073.94	**1064.74**	1065.46
	2–3	1028.58	994.73	982.53	**982.14**
	3–4	748.94	735.64	732.58	**728.38**
Raw materials	1–2	1739.98	1640.87	**1635.12**	1650.37
	2–3	1565.29	**1488.80**	1494.98	1495.52
	3–4	1286.09	1275.26	**1246.95**	1247.57

The bold numbers represent the smallest AIC values in the different distributions

### Analysis of Multistate Influencing Factors

The results of the accelerated failure time model for the non-manufacturing and manufacturing enterprises in different states are provided in Tables 4 and 5. At Trans. 1–2, for non-manufacturing enterprises, the supply shortages, cash flow shortages, order cancellations, and employee panic have significant effects on the initial recovery time at the 5% significance level. The acceleration factor of employee panic is exp(0.257) = 1.293, which means it leads to a 1.29 times longer recovery time. We define the other variables as constant and consider the initial recovery time of enterprises without employee panic as the baseline recovery time, which is estimated to be 27.856 [25.158, 30.576] days, while the recovery time of enterprises with employee panic is 36.014 [32.790, 39.429] days. By analogy, the acceleration factors for supply shortages, cash flow shortages, and order cancellations are 1.081, 1.323, and 1.067, respectively. At Trans. 2–3, supply shortages and order cancellations still hinder enterprise recovery, while employee panic and cash flow shortages no longer affect mid-term recovery, which is in turn affected by employee shortages, with an acceleration factor of 1.133. Since the employee shortage variable was preprocessed by logarithmic transformation, a 10% increase in employee shortages implies that enterprise recovery time is extended by around 1.133 × ln(1.1) = 10.79%. At Trans. 3–4, the factors affecting enterprises’ full recovery are the same as those affecting mid-term recovery. By analyzing the recovery states of enterprises and their determinants, in the Covid-19 pandemic context, order cancellations and supply shortages affect the entire recovery process, while cash flow shortages and employee panic only affect initial recovery. The emergence of employee shortages has hindered mid-term and full recovery.

**Table 4:** Non-manufacturing recovery state estimates

Variables	Trans. 1–2		Trans. 2–3		Trans. 3–4	
	Est.	Std.	Est.	Std.	Est.	Std.
*Non-manufacturing*						
Customer distribution (outside the province)	0.127^•^	0.070				
Supply shortages (log-scale)	0.078***	0.021	0.074**	0.025	0.100*	0.046
Cash flow shortages (with)	0.280**	0.089				
Employee shortages (log-scale)			0.125***	0.024	0.116**	0.045
Employee panic (with)	0.257***	0.059				
Order cancellations (log-scale)	0.065***	0.017	0.096***	0.021	0.158***	0.032
*Services*						
Supply shortages (log-scale)	0.066*	0.026	0.088*	0.035		
Cash flow shortages (with)	0.320**	0.112	0.240*	0.119		
Employee shortages (log-scale)			0.118***	0.029		
Employee panic (with)	0.261***	0.076				
Order cancellations (log-scale)	0.105***	0.021	0.104***	0.026	0.111**	0.042
*Wholesale and retail*						
Customer distribution (outside the province)	0.312**	0.108	0.364**	0.139	0.515**	0.195
Number of employees (log-scale)	− 0.093**	0.033	− 0.087*	0.040	− 0.132*	0.055
Supply shortages (log-scale)					0.314***	0.071
Employee shortages (log-scale)	0.142***	0.032	0.222***	0.044		
Employee panic (with)	0.291**	0.101				
Order cancellations (log-scale)			0.075^.^	0.044	0.262***	0.056

*Est.* Estimated, *Std.* Standard error, *Trans.* Transition

^•^0.05 < *p* < 0.1; *0.01 < *p* < 0.05; **0.001 < *p* < 0.01; ***0 < *p* < 0.001

**Table 5:** Manufacturing recovery state estimates

Variables	Trans. 1–2		Trans. 2–3		Trans. 3–4	
	Est.	Std.	Est.	Std.	Est.	Std.
*Manufacturing*						
Business ownership (state-owned businesses)	− 0.243^•^	0.147				
Raw material shortages (log-scale)					0.094**	0.032
Employee shortages (log-scale)			0.119***	0.022		
Employee panic (with)	0.119*	0.057				
Order cancellations (log-scale)	0.055***	0.016	0.050*	0.027		
*Livelihood-related*						
Number of employees (log-scale)			− 0.098^•^	0.053	− 0.126^•^	0.069
Raw material shortages (log-scale)					0.124^•^	0.067
Employee shortages (log-scale)			0.106*	0.053		
Order cancellations (log-scale)	0.079^•^	0.040	0.116^•^	0.061		
*Processing and assembly*						
Raw material shortages (log-scale)					0.139*	0.062
Employee shortages (log-scale)			0.161***	0.034		
Order cancellations (log-scale)	0.050*	0.024				
*Raw material*						
Business ownership (state-owned businesses)	− 0.434*	0.175	− 0.553**	0.201		
Employee shortages (log-scale)			0.068*	0.032		
Employee panic (with)	0.213**	0.083				
Order cancellations (log-scale)	0.057*	0.024	0.088**	0.030		

*Est.* Estimated, *Std.* Standard error, *Trans.* Transition

^•^0.05 < *p* < 0.1; *0.01 < *p* < 0.05; **0.001 < *p* < 0.01; ***0 < *p* < 0.001;

An industry breakdown of non-manufacturing enterprises reveals the determinants of the different recovery states of the service industry and wholesale and retail industry enterprises exhibiting characteristics specific to their industry activities. For service industry enterprises, order cancellation issues are key to recovery, while other issues disappear as the enterprise gradually recovers. For wholesale and retail industry enterprises, customer distribution influences all recovery stages. Enterprises selling products to customers mainly located outside their provinces recover more slowly than those whose customers are located inside the province, probably due to the strict control measures and inconvenient transportation caused by the pandemic. The wholesale and retail industry also depend on the number of employees, with an acceleration factor of exp(− 0.132) = 0.876 at Trans. 3–4, which indicates that a higher number of employees is linked to faster recovery.

Manufacturing enterprises have more complex recovery determinants in different states because of the large variability in their products. The acceleration factor of employee panic is 1.126, which means that the recovery time of the enterprises experiencing employee panic is 1.13 times longer than that of enterprises not experiencing this problem. Similarly, the acceleration factor of order cancellations is 1.057, which means that the recovery time of enterprises with a 10% increase in order cancellations is increased by around 1.057 × ln(1.1) = 10.07%. At Trans. 2–3, the mid-term recovery determinants are employee shortages and order cancellations, with acceleration factors of 1.125 and 1.051, respectively. For example, while holding other variables constant, the expected mid-term recovery time with employee shortages is 31.141 [28.984, 33.611] days. At Trans. 3–4, raw material shortages become the main determinant of full recovery, with an acceleration factor of 1.098. The initial recovery of manufacturing enterprises is mainly affected by employee panic and order cancellations, which are closely related to the severity of the initial Covid-19 crisis. Employee shortages become the main factor that hinders the recovery of enterprises in the mid-term. When manufacturing enterprises have recovered to a certain level, the shortage of raw materials constrains full recovery.

The processing and assembly, and livelihood-related manufacturing sectors broadly conform to the above characteristics. However, for raw material manufacturing, business ownership has a significant effect on the initial and mid-term recovery of enterprises. The acceleration factor for state-owned enterprises at Trans. 2–3 is exp(-0.553) = 0.575, which implies their recovery is faster than that of non-state-owned enterprises.

### Transition Probabilities

The accelerated failure time model evaluates the factors that affect the recovery time of an enterprise in a given state and, based on this, the multistate model measures the probability that such factors change the enterprise recovery state. Using flexsurv—a statistical analysis package in R language—we calculated the transition probabilities between different recovery states for the non-manufacturing and manufacturing industries (Jackson 2016).

#### State Transition Probability of Enterprise Recovery without Introducing Covariates

**Fig. 2:**
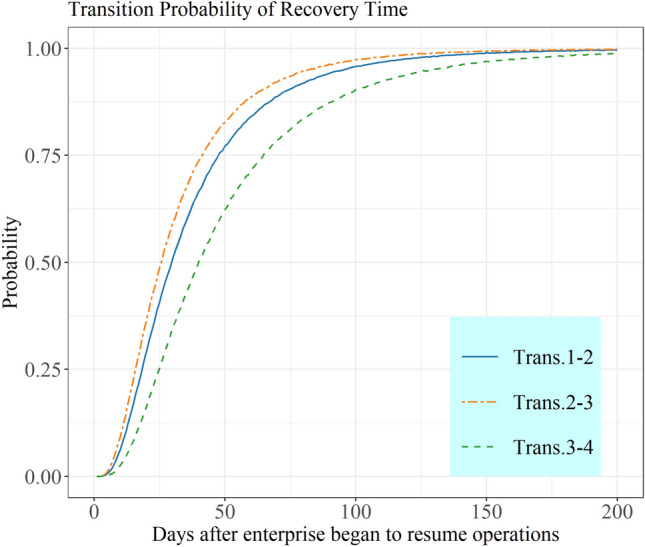
State transition probability. Trans. 2–3 indicates that the enterprise reaches the mid-term recovery state after the initial recovery state, namely, Trans. 1–2–3; Trans. 3–4 indicates that the enterprise reaches the full recovery state after the initial and mid-term recovery state, namely, Trans. 1–2–3–4

The state transition probability of enterprise recovery without introducing covariates is estimated by multistate model. Figure 2 shows the transition probability of enterprises reaching different recovery states after they began to resume operations, which is the estimation of the sample population. The results reveal the general trend of enterprise recovery.

The shock on Chinese enterprises in the early stages of the pandemic was enormous. Many of them were not able to recover their operation in the face of strict controls and severe market conditions. In response to the potential economic crisis, the government introduced supportive policies to help enterprises recover, and the instantaneous recovery rate of enterprises showed an upward trend as a result. In the middle and later stages of the pandemic, since enterprises had recovered to a certain level and full recovery could not be accomplished in the short term, the instantaneous recovery rate began to decline. This estimate is also consistent with the log-normal hazard function used to evaluate the recovery process of enterprises, and it also verifies that the instantaneous recovery rate during the recovery process increased to a peak and then gradually decreased.

#### Recovery State Transition Probability for Non-manufacturing Enterprises by Introducing Covariates

**Fig. 3:**
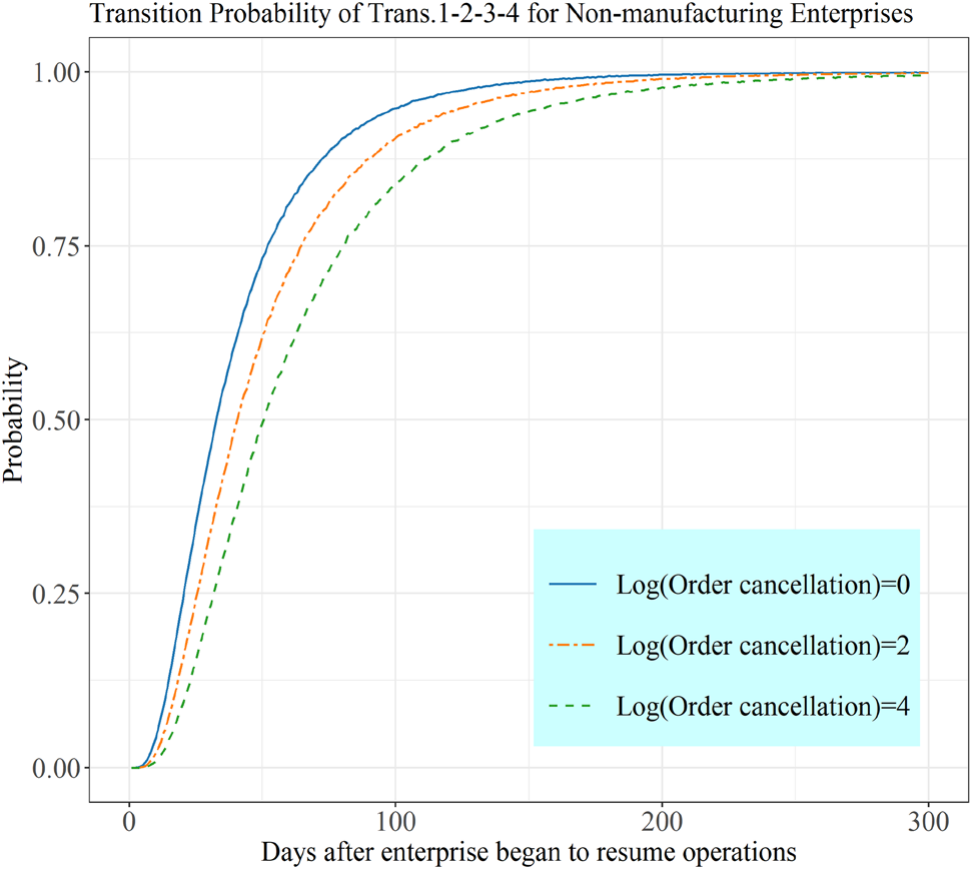
Effect of order cancellations on the state transition probability for non-manufacturing enterprises

The results of the multistate model (see Tables 4 and 5) show that the covariates have a significant effect on recovery time at different stages. For non-manufacturing enterprises, order cancellations and supply shortages are the main determinants of enterprise recovery. Taking order cancellations as an example, the effect of order cancellations on the transition probability from no recovery to full recovery for non-manufacturing industries is shown in Fig. 3. The data are preprocessed by logarithmic transformation for increased stability, while maintaining the trend of the distribution. The effect of order cancellations, two months after enterprises began to resume operations, on the transition probability of enterprises were studied. When the logarithms of order cancellation are 0, 2, and 4, the probabilities of moving to a full recovery state are 0.815, 0.716, and 0.607, respectively, which means that order cancellation with an exp(2) increase will lead to around 10% increases in transition probability.

Service industry enterprises have the same characteristics as non-manufacturing enterprises, meaning order cancellations still hinder their recovery states. Moreover, cash flow shortages have a more significant impact on the recovery time of service industry enterprises in the initial and mid-term stages.

**Fig. 4:**
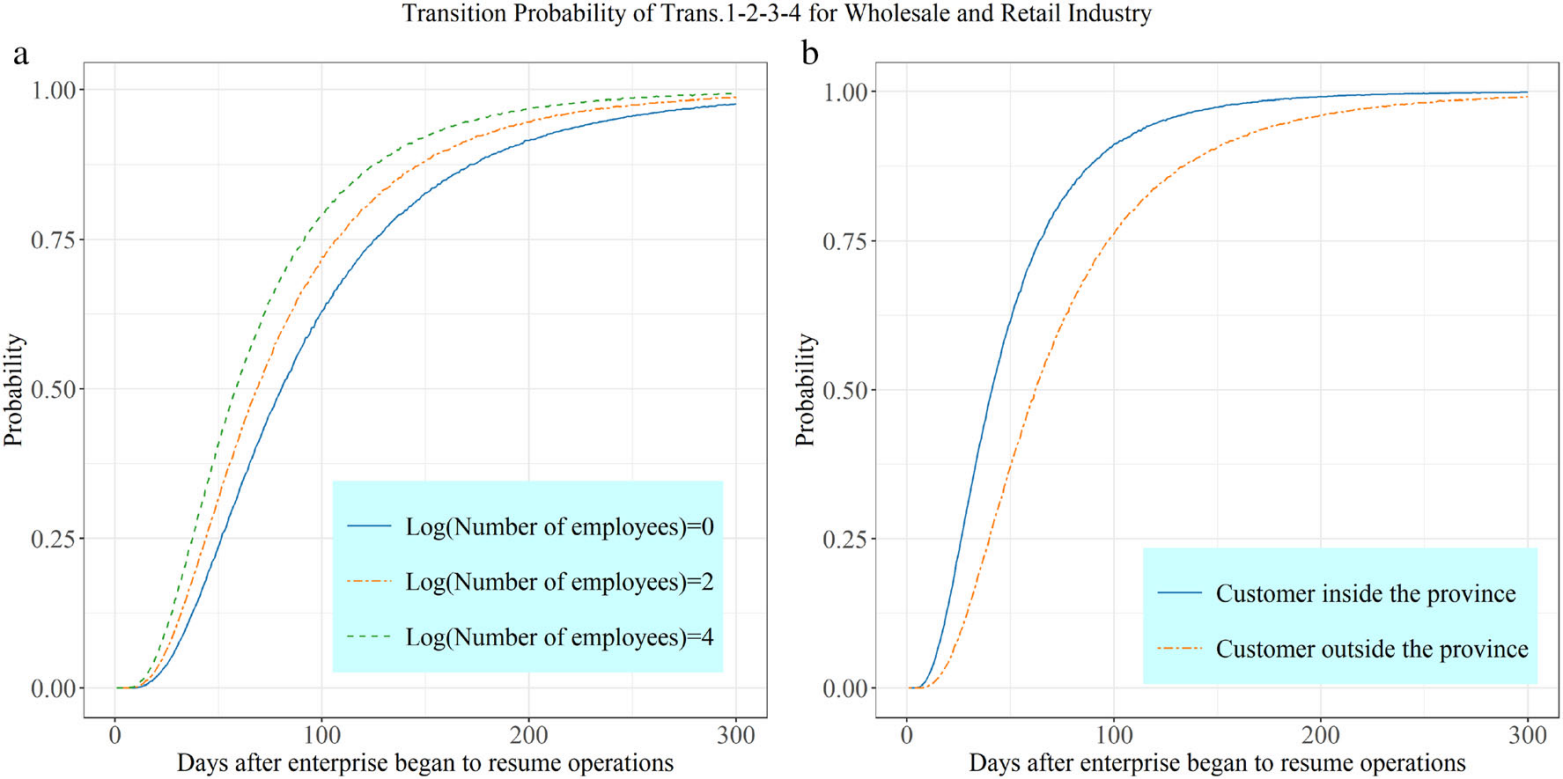
Effects of typical explanatory variables on the state transition probability for the wholesale and retail industry

Compared with the service industry, the recovery process is more demanding for the wholesale and retail industry in terms of the customer distribution and number of employees. Figures 4a and b illustrate how the number of employees and customer distribution affect the transition probability from no recovery to full recovery. Figure 4a shows that the higher the number of employees, the higher the transition probability of an enterprise when holding other covariates constant. Figure 4b shows that enterprises whose customers are mainly located in the province, compared to those whose customers are located outside the province, have a higher transition probability, and the difference between them is significant. This indicates that customer distribution is a main determinant of the recovery process of wholesale and retail enterprises. Customer distribution two months after enterprises began to resume operations indicates a transition probability of 0.726 and 0.487 for enterprises with customers in and outside the province, respectively. The above analysis shows that, for the non-manufacturing industry and its subsectors, the main factors that hinder recovery in the Covid-19 context are the movement of personnel, capital, and market demand.

#### Recovery State Transition Probability for Manufacturing Enterprises by Introducing Covariates

**Fig. 5:**
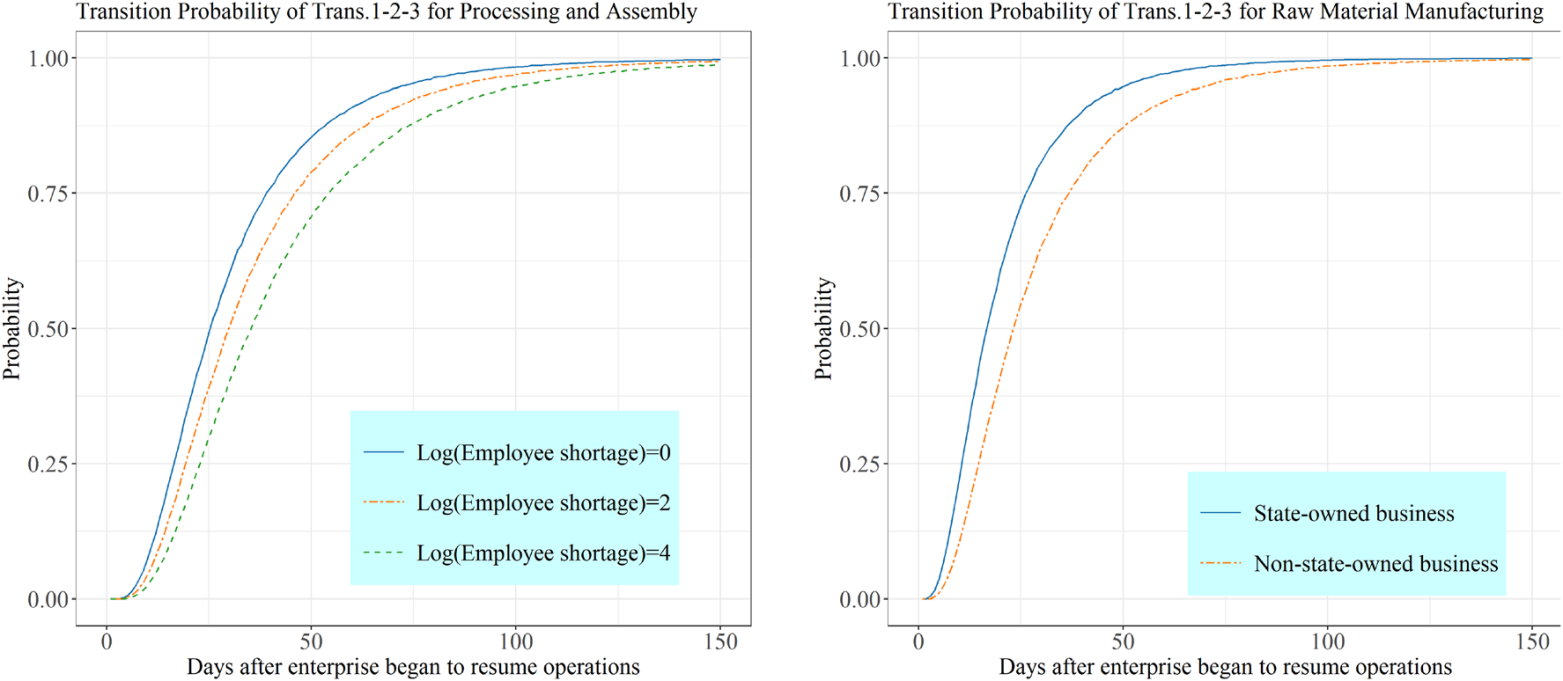
Effects of typical explanatory variables on the state transition probability for the processing and assembly and raw material manufacturing industries

Different factors affect the recovery of manufacturing enterprises at different stages, with order cancellations and employee panic being the main determinants of the initial recovery process, while employee shortages become an obstacle for mid-term recovery and raw material shortages are a constraint to full recovery. Taking processing and assembly enterprises as an example, Fig. 5a shows the probability of moving from no recovery to mid-term recovery for different employee shortage levels. Same as the analysis of non-manufacturing enterprises, we focused on the effect of employee shortages on the transition probability of enterprises and recovery time. The transition probabilities to a mid-term recovery state are 0.913, 0.866, and 0.802 for two months after enterprises began to resume operations and employee shortage logarithms of 0, 2, and 4, respectively, which means that the transition probability from no recovery to mid-term recovery decreases by around 5% as the employee shortage percentage has an exp(2) increase.

Unlike other manufacturing enterprises, the impact of enterprise ownership on the transition probability is greater for the raw material manufacturing industry, and Fig. 5b shows that state-owned enterprises have a higher transition probability from the no recovery to the mid-term recovery state than non-state-owned ones. For instance, the transition probabilities for state-owned and non-state-owned enterprises are 0.973 and 0.925 in two months after enterprises began to resume operations, respectively. The analysis of the manufacturing sector and its subsectors reveals that the pandemic mainly affected the recovery of manufacturing enterprises in terms of personnel and raw materials.

## Discussion

The Covid-19 pandemic has become an epochal global public health event, and the contagiousness and diffusion of this virus have had different impacts on national and global societies from those of natural hazards and disasters that are more localized. This has led to a financial crisis with different characteristics than those of crises induced by natural hazards and disasters. Specifically, natural hazards and disasters are more likely to destroy the physical infrastructure on which enterprises highly depend, resulting in reduced production capacity. In contrast, the loss caused by Covid-19 to enterprises is reflected more in the imbalance of the economic system, such as reduced consumption, unemployment and re-employment difficulties, and supply chain disruptions (Coibion et al. 2020; Lu et al. 2020; Nicola et al. 2020). The recovery of enterprises after natural hazards and disasters follows a more Weibull type distribution (Yang et al. 2016), while in the case of Covid-19 a log-normal distribution is more suitable for the data from manufacturing and non-manufacturing industries and their subindustries, reflecting enterprise recovery rates.

The assumption of the time multiplicative effect under the accelerated failure time model forms the basis of our research. The acceleration factor is an important indicator of the time multiplicative effect, based on which the baseline recovery time of enterprises without the influence of this factor can be compared with the baseline recovery time under the influence of the factor. However, an enterprise’s dynamic recovery process comprises single or multiple events with time-varying covariates. The survival analysis model can be used to model the time of the first event, but the subsequent events will be ignored (Crowther and Lambert 2017). Therefore, this study proposed a multistate framework, which treats the enterprise recovery process as a series of discrete events and solves the problem of time-varying heterogeneity of data. The empirical results for non-manufacturing industries (see Sect. 3.2) also reflect the intrinsic event trend, that is, the recovery of the service industry depends on increases in orders, while the recovery of the wholesale and retail industry depends more on the number of employees and customer distribution, which is consistent with Yang et al.’s (2021) and Liu et al.’s (2021) findings. The transition probability (see Sect. 3.3) reflects the possibility of dynamic changes in the recovery state of an enterprise under the influence of a certain factor. The application of the multistate and accelerated failure time models to enterprise recovery time can provide theoretical references for decision makers’ emergency management and, at the same time, draw on the recovery experience of Chinese enterprises to help foreign enterprises that have not yet recovered assess their state and formulate coping strategies.

The Covid-19 crisis has had a significant impact on the production and operation of enterprises, and this study examined the factors that have affected enterprise recovery during the pandemic and the transition between different recovery states. The results present the recovery process of enterprises in different industries during the pandemic. However, this study has some limitations. Since it is a data-driven empirical analysis, the interpretation of the results relies entirely on the collected data. Insufficient interpretation of the analysis results is also possible due to data sampling errors, which are mainly reflected in two aspects. On the one hand, there is only one variable for the livelihood-related manufacturing industry that affects the mid-term recovery of enterprises at the 5% significance level. On the other hand, there is no raw material shortage problem throughout the recovery process for the raw material manufacturing industry, which is also worth discussing. Therefore, future studies should verify the reliability of manufacturing data based on the scientific design of the sampling method, reduce data errors through multiple sampling surveys, and thus optimize the analysis results for the manufacturing data.

## Conclusion

The purpose of this study was to examine the recovery process of enterprises in the context of the Covid-19 pandemic and apply the multistate model to explore the effects of time-varying covariates on the different enterprise recovery stages. Based on the above analysis, we can draw the following conclusions:

1. A combination of the accelerated failure time and multistate models is suitable for studying the dynamic recovery process of enterprises. The factors affecting the recovery time of an enterprise are identified using survival analysis, and the superiority and applicability of this method for the duration analysis are demonstrated. The dynamic recovery process of enterprises is depicted using the multistate model, which comprehensively reflects how internal and external factors affect recovery under the Covid-19 scenario.
2. The accelerated failure time model can capture the determinants of the different recovery stages and deepen our understanding of the enterprise recovery process. For non-manufacturing industries, the determinants of recovery always apply to multiple or all stages of recovery. However, for manufacturing industries, initial recovery is mainly a matter of order cancellations, mid-term recovery faces employee shortages, and full recovery depends on the adequacy of raw materials.
3. The enterprise recovery process is divided into discrete states, and the dynamic transition probability between the states is measured by a multistate model based on the semi-Markovian assumption. Combined with the variables identified by the accelerated failure time model, the changes in the state transition probability of enterprises under the influence of this variable are analyzed. When the degree of negative influence is greater, the transition probability for the same time scale is greater. For the influence of the number of employees on the full recovery phase of the wholesale and retail industry (see Fig. 4a), when the positive influence is greater, the transition probability of enterprises is smaller, such as for the influence of order cancellations on the full recovery phase of the non-manufacturing industry (see Fig. 3). Most variables in this study show positive influences, but less consideration is given to the contribution of favorable conditions within and outside the context of enterprise recovery time. Therefore, incorporating more variables to consider enterprise recovery in the pandemic context in an integrated manner could further improve our results.
4. By using empirical data, this study can provide a theoretical reference for enterprise managers to address business survival. For example, the traffic ban introduced by the government during the Covid-19 pandemic impeded the flow of customers, and the offline sales channels of non-manufacturing industries, which strongly depended on customer flow, were blocked from operating normally. However, enterprises could change their sales patterns and develop online sales channels to adapt to these exogenous changes. Due to the high cost of sample data collection, this study did not analyze other industry segments. Limited by selected indicators, factors affecting enterprise recovery were not considered comprehensively. Therefore, controlling the sampling method, expanding the sample size, and comprehensively considering other industry sectors and the determinants of enterprise recovery are key elements to expand this study.

## Footnotes

1 https://www.wenjuan.com/.

2 https://www.tianyancha.com/.
